# Telemedicine improves mental health in COVID-19 pandemic

**DOI:** 10.7189/jogh.11.03004

**Published:** 2021-03-07

**Authors:** Md Yeasin Arafat, Sanjana Zaman, Mohammad Delwer Hossain Hawlader

**Affiliations:** 1 Department of Public Health, North South University (NSU), Dhaka, Bangladesh; 2 Department of Public Health, Daffodil International University, Dhaka, Bangladesh

The gruesomeness and the negative impact of the COVID-19 situation, particularly on the physical and mental health of the people know no bounds. On 11 March 2020, the World Health Organization has declared this as a pandemic [1,2]. Several countries introduced meticulous steps to restrain physical movement as part of efforts to reduce the infection rate of COVID-19, more and more of us are making considerable changes to our regular lifestyle [3]. People have been facing the new realities of home office, temporary unemployment, home-schooling of children, and keep social distancing with other family members, friends and colleagues take time to get habituated [4]. The repercussions of such episodes not as it negatively affects physical but mental well-being as well [5]. However, it has been seen that with the help of telemedicine, this burden can be reduced as it is convenient during this pandemic situation [6].

The World Health Organization has defined telemedicine as the delivery of health care services at a distance using electronic implies for “the diagnosis of treatment, and prevention of disease and injuries, research and evaluation, education of health care providers” to improve well-being [7]. Telemedicine through telephone and video technology has been used since the 1960s in the sectors of the military and space and its use has been increased significantly in the past decades [7]. However, Pandemics and other public health emergencies typically lead to a surge in demand for medical care, which overwhelms local capabilities [8]. During this COVID-19 situation, it is well documented that to provide rapid access to specialists who are unavailable in person and precisely in non-surgical cases, telemedicine provides the best treatment option.

From our knowledge, telemedicine makes mental health services more accessible as it helps patients avoid stigma, and get treatment from the privacy of home. Different mental health problems typically anxiety, depression and substance-related disorder, etc. can be treated with telemedicine efficiently and effectively, particularly in the primary health care setting [9]. For instance, in America, it is seen that telemedicine has tremendous potential to improve the lives of patients suffering from depression and other mental health issues [10]. Again, telemedicine is also helpful to treat anxiety after stroke/TIA [11]. Moreover, Telepsychiatry to a rural Geri psychiatric inpatient unit yielded positive results in terms of satisfaction compared with in-person care [12]. Therefore, from our perspective, maybe there are some shortcomings of telemedicine in treating critical mental health issues that we cannot mention precisely. Still, we firmly believe that telemedicine provides a handy link for patients with special requirements, including the young, minority populations, and the elderly.

COVID-19 has caused severe psychological impact by causing anxiety, phobia, paranoia, hoarding, posttraumatic stress disorder (PTSD), depression, mass hysteria, economic burden, and financial losses. These psychological burdens occur due to early lockdown, isolation and social distancing, and these incidences result in boredom and loneliness. A recently published paper from China mentioned that the various countries across the world such as the Chinese, Singaporean, and Australian governments, concern about the psychological health of the people during this pandemic situation and its long-term effect. To prevent this issue, they have implemented telemedicine facilities through videoconference, e-mail, telephone and smartphone apps. Telemedicine was shown to be feasible, acceptable, and effective in Western China, and allowed for significant improvements in health care outcomes [6].

In the past years, it is seen that telemedicine has had a positive outcome for treating numerous epidemic diseases and played a significant role in treating health problems like psychological distress, depression, insomnia and other mental health disorder [13]. For example- in 2014-2016, Africa was combating the Ebola crisis by using a mobile app named Ebola Contact Tracing (ECT). Again, in 2003 teleconsultation was used in Taiwan to treat severe acute respiratory syndrome (SARS). The Swiss Centre for Telemedicine, Medgate was effective against influenza-like symptoms in Switzerland [14]

A recently published paper from Lebanon depicts that in this pandemic situation the level of stress has greatly increased in both symptomatic and asymptomatic COVID-19 affected people · Moreover, prior mental health affected the patient’s condition is disproportionately affected since they are more vulnerable to stress and panic. In the solution, the authors suggest that, to solve this problem public health recommendations and telemedicine need to be implemented in a widespread manner [15]. Another recently published paper mentioned, both COVID-19 infected and non-infected people are equally prone to have mental health disorders during this pandemic situation. To manage this problem, they suggest that telemedicine is the ideal choice and it should practice not only in the emergency condition but also in usual practice [16].

The advantages of telemedicine in this pandemic situation have been well documented. Telemedicine can support long-distance clinical care, education, and health administration. The patients who need care for anxiety and depression can be assisted without the requirement for visiting a hospital, and therapy for psychological stabilization can be provided via the internet, without the typical face to face visit with the doctor. Moreover, by decreasing the number of clinic visits for periodical discussions and medicines among the geriatric populace with mental ailments, telemedicine may possibly decrease the number of secondary or tertiary diseases. Furthermore, telemedicine may also reduce the loss of follow-up among psychiatric patients [17]. It is also helpful for improved professional education, quality control of screening programs, reduced health care costs, and improved access to information [18].

**Figure:**
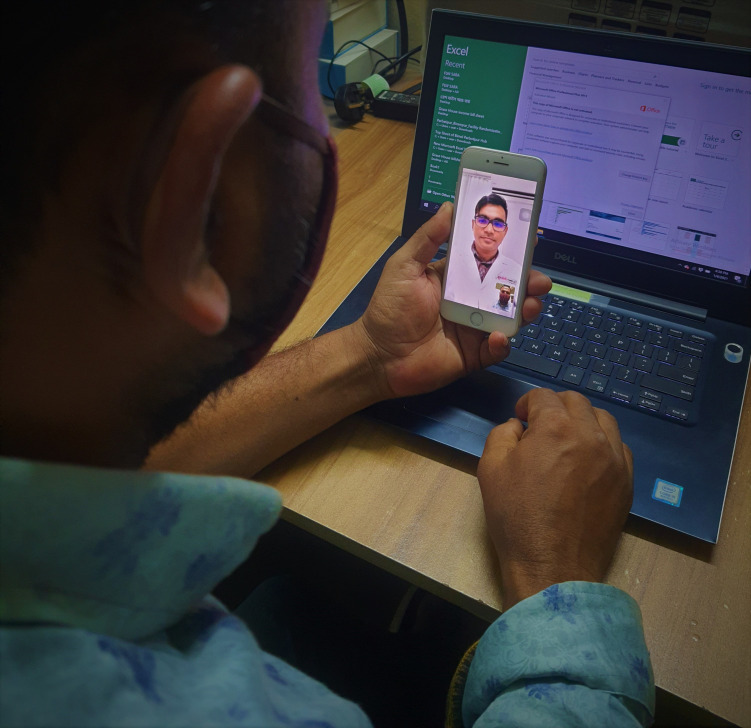
Photo: From the author’s own collection, used with permission.

However, there are numerous challenges to its execution. For example, the Federal Medical Council of Brazil has regarded the practice of telemedicine (between patient and doctor) as not lawful [19]. Likewise, in most states in the USA, the utilization of telemedicine is extremely compelled by administrative bodies. Technologies for e-health care system require satisfactory transmission capacity to bolster the transmission of information, pictures and sound. Consequently, access to broadband internet connection is a must for telemedicine in e-health care. This factor increases difficulties for the rural people, those without an internet connection, or underprivileged groups who cannot afford this service [20]. It is also reported that, the main drawbacks of telemedicine are a breakdown in the relationship between health professionals and their patients; a breakdown in the relationship between health professionals; issues regarding the quality of health information data; and organizational and bureaucratic challenges [21]. Indeed, in spite of the fact that the technology is now not an issue in this day and age for telemedicine to be broadly utilized, direction and cybersecurity issues stand as a challenge for its ceaseless selection.

In this current situation, the two most crucial considerations are the quality of the telemedicine service delivered, and its safety [22]. As the telemedicine revolution is gaining exponential momentum, the practices embraced amid this pandemic will resonate all through the lobbies of clinics and hospitals for the predictable future. Once the telemedicine's patients, providers, administrators, and policymakers see that this model works, it cannot be undone [23]. Therefore, tele-mental health might be the option to alleviate the risk of physicians or patients being infected while still providing treatment, especially where there is a scarcity of psychiatrists and other mental health professionals [15]. Again, while private insurers remain interested in telehealth, it may not be a boon for private practices, even if they decide to continue reimbursement after the public health emergency ends [24].

Telephone-delivered psychotherapy has increased utility as a method of service delivery in the current world, where several barriers, including economic hardships and limited access to care, may prevent people from receiving the treatment they need [25]. It refers to the provision of psychotherapy services using telecommunication technologies including email, text messaging, video conferencing, online chat, messaging, or internet phone [26]. Again, it offers increased client convenience concerning the location and flexible timing of appointments [25]. Tele-psychotherapy sessions are to be conducted on an appointing basis, and it needs a couple of sessions. These sessions are strictly abiding by confidentiality from both sides (doctors and patients). Insurance coverage for telemedicine is affected by government and state laws as well as Insurance company approaches. In spite of the fact that a few are more dynamic than others, these days’ numerous state councils and private health insurance suppliers are recognizing the potential of telemedicine to decrease costs and keep patients healthier. The federal government is also looking forward to using telemedicine in a widespread manner under Medicare [27].

From the aforementioned discussions, it would not be deemed dare to say that, in spite of having some limitations, telemedicine is perfectly suitable to treat the mental health problems of the people in this pandemic situation without increasing the risk of infection, promoting health and prolonging life as well. In our opinion, telemedicine is a major step forward for the people as it is safer, useful, and more convenient in order to obtain a sound mind and sound health.
